# Supplementary figures and images for: Fungal Footprints: Soil Fungal Communities in Black Walnut and Red Oak Forests
Source: Microorganisms. 2024 Oct 30;12(11):2184. doi: 10.3390/microorganisms12112184 (PMC11596218; doi:10.3390/microorganisms12112184)

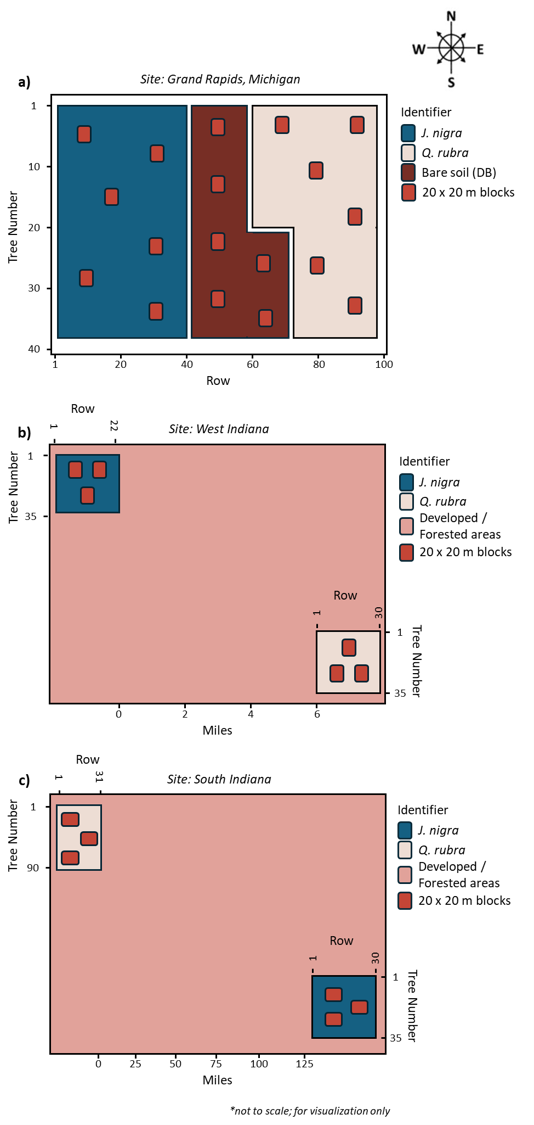

Supplement: Supplementary file 1 [file microorganisms-12-02184-s001.zip › microorganisms-3257738-supplementary.png]
